# A Conceptual Framework for the Assessment of Cumulative Exposure to Air Pollution at a Fine Spatial Scale

**DOI:** 10.3390/ijerph13030319

**Published:** 2016-03-15

**Authors:** Kihal-Talantikite Wahida, Cindy M. Padilla, Zmirou-Navier Denis, Blanchard Olivier, Le Nir Géraldine, Quenel Philippe, Deguen Séverine

**Affiliations:** 1 School of Public Health (EHESP), Rennes, Cedex 35043, France; Wahida.kihal@ehesp.fr (K.-T.W.); Cindy.padilla@ehesp.fr (C.M.P.); denis.zmirou@inserm.fr (Z.-N.D.); Olivier.Blanchard@ehesp.fr (B.O.); Philippe.QUENEL@ehesp.fr (Q.P.); 2 INSERM U1085 IRSET (Research Institute in Environmental and Occupational Health), Rennes, Cedex 35000, France; 3 Medical Department, Lorraine University, Nancy, Cedex 54052, France; 4 Air Quality Monitoring Associations (AASQA), Airparif, Paris 75004, France; Geraldine.LeNir@airparif.asso.fr

**Keywords:** air pollution, long-term, cumulative exposure assessment, residential mobility, fine spatial scale

## Abstract

Many epidemiological studies examining long-term health effects of exposure to air pollutants have characterized exposure by the outdoor air concentrations at sites that may be distant to subjects’ residences at different points in time. The temporal and spatial mobility of subjects and the spatial scale of exposure assessment could thus lead to misclassification in the cumulative exposure estimation. This paper attempts to fill the gap regarding cumulative exposure assessment to air pollution at a fine spatial scale in epidemiological studies investigating long-term health effects. We propose a conceptual framework showing how major difficulties in cumulative long-term exposure assessment could be surmounted. We then illustrate this conceptual model on the case of exposure to NO_2_ following two steps: (i) retrospective reconstitution of NO_2_ concentrations at a fine spatial scale; and (ii) a novel approach to assigning the time-relevant exposure estimates at the census block level, using all available data on residential mobility throughout a 10- to 20-year period prior to that for which the health events are to be detected. Our conceptual framework is both flexible and convenient for the needs of different epidemiological study designs.

## 1. Introduction

The World Health Organization has estimated that deaths attributed to outdoor air pollution are predominantly due to ischemic heart diseases and strokes (80%), followed by chronic obstructive pulmonary diseases or acute lower respiratory infections (14%) and lung cancer (6%) [1].

Several original papers, reviews and meta-analyses have documented that mortality (including mortality from all-causes) and chronic diseases (especially cancer, cardiovascular and respiratory disease) are associated with long-term exposure to particulate air pollution—notably to particles with diameters of 10 µm or less (PM_10_), and particles with diameters of 2.5 µm or less (PM_2.5_) and their constituents [2,3,4,5], as well as to nitrogen dioxide (NO_2_) [2,4,5,6,7] and sulfur dioxide (SO_2_) [2,4,5,6,7,8]. Regarding all-cause mortality, these studies have revealed an increase in mortality with long-term exposure to elemental carbon (pooled estimate: +6% per 1 μg/m^3^; CI95% (5%−7%)), NO_2_ (pooled estimate: +5% per 10 μg/m^3^; CI95% (3%−8%)), and PM_2.5_ (pooled estimate: +6% per 10 μg/m^3^; CI95% (4%−8%). Mortality from cardiovascular and non-malignant respiratory diseases was significantly associated with long-term exposure to NO_2_ (pooled estimate:+11% per 10 μg/m^3^;CI95% [5%–16%]; +3% CI95% (6%−13%) respectively) [4]. Recently, Bentayeb *et* *al.* have shown that long-term exposure to PM_10_, PM_2.5_, NO_2_, SO_2_ and benzene is strongly associated with an increased risk of non-accidental mortality in France [7].

A Scientific Statement from the American Heart Association supports that long-term exposure to particulate matter has cardiovascular effects [9,10]. Regarding cancer, the International Agency for Research on Cancer (IARC) has classified air pollution, as a whole, as carcinogenic to humans, and had already determined that several components of outdoor air pollution were carcinogens, including diesel engine exhaust, solvents, metals, and dust [11].

Compared to the health effects of short-term exposures, the impact of air pollution on chronic diseases has given rise to a more limited body of literature. Since the seminal publications of the Harvard Six Cities Study, which examined the relationship between long-term ambient concentrations of particulates and mortality in a cohort of about 8000 adults between 1976 and 1989 [12], between 1979 and 1998 [13], and between 1974 and 2009 [3], results of other important cohort studies have been published. Most were performed in the U.S., such as the American Cancer Society study [14,15,16], the Women’s Health Initiative Observational Study [17], the Nurses’ Health Study [18] and the Medicare national cohort [19]. In Europe, the results of a growing number of studies were published, including those of the Netherlands cohort study [20], the Rome longitudinal study [21], the ESCAPE Project (European Study of Study of Cohorts for Air Pollution Effects) [22], the French PAARC survey [6] and the Gazel cohort study [7].

All these studies concluded that long-term exposure has a positive association with chronic diseases. Case-control studies in Canada revealed that breast cancer was associated to long-term exposure to NO_2_ (OR = 1.32; CI95% (1.05−1.67) for a 10 ppb increase in NO_2_ [23]).

The Women’s Health Initiative cohort study [18] and 11 European cohorts within the ESCAPE Project [22] found an increase of the risk of stroke incidence with the annual concentrations of PM_2.5_ (+19% per 5 μg/m^3^ increase in PM_2.5_ [22] and +28% among women per 10 μg/m^3^ increase in PM_2.5_ [17], respectively. Also, the U.S. study cohort shows that long-term exposure to PM_10_ was associated with elevated risks of incident stroke (OR = 1.06; CI95%, (1.00–1.13)) [24].

One common limitation of these epidemiological studies on the effects of long-term exposure to air pollution lies in the assessment of cumulative exposure. One aspect deals with the choice of the exposure metric when striving to reconstruct cumulative exposure over latency periods of, say, 20 years, in the course of which exposure levels may vary. Over such time periods, individual assessment of exposure cannot apply, irrespective of the study design. Some form of a semi-ecological approach based on spatial patterns of air quality has to be used, following several approaches [25] including data retrieved from monitoring stations [12,14,23,26], geostatistical modeling [23], and retrospective dispersion modeling [27].

These approaches lend themselves to a form of exposure misclassification arising out of the spatial heterogeneity of pollutant concentrations dependent on their sources, the density of monitoring stations in the study areas, and dispersion factors (both meteorological and topographical characteristics). This heterogeneity varies across air quality indicators (it is greater for NO_2_ than for PM_2.5_, for example) and size of the spatial scale of the analysis (the finer the spatial scale, the lower the heterogeneity). The degree of spatial resolution of air concentrations of pollutants in the different assessment approaches may lead to substantial exposure misclassification, which results in biases in the estimations of the concentration-response functions.

A second form of misclassification relates to failure to consider temporal and spatial mobility of subjects, which essentially has two aspects. One results from short-term mobility patterns; people are exposed to air pollutants at their place of residence and, depending on their activity profile, at the workplace or when they are out shopping or commuting between these locations. The other aspect results from long-term residential mobility patterns that describe the geographic mobility of the population during the study follow-up, when exposure is assessed over long periods. Because of the potential influence of residential mobility, its consideration in cumulative exposure assessment is a crucial component for all epidemiological study designs exploring the effects of long-term exposures.

### The Purpose of This Paper

To our knowledge, no epidemiological study investigating the health effects of long-term exposure to air pollution has accounted for residential mobility in assessing cumulative exposure at a fine spatial scale. This paper is an attempt to fill this gap via the development of a conceptual model for the assessment of cumulative exposure to air pollution at a fine spatial scale. Our work is part of a project that aims to investigate the long-term effects of air pollution on breast cancer in Paris, France, using an ecological approach whereby all data (regarding outcome, exposure, confounders or effect modifiers) will be collected at the smallest geographical level currently available in France, namely census block level (known as “IRIS”—a French acronym for “blocks for incorporating statistical information”).

The present paper comprises four sections:

(i) We present an overview of exposure assessment in the exploration of long-term effects in epidemiological studies.
(ii) We propose a conceptual framework for the retrospective assessment of air pollution, including two components:
  (1) retrospective reconstitution of NO_2_ concentrations at a fine spatial scale (NO_2_ has been chosen as the index pollutant because its spatial variability is higher than in many other air pollutants); and
  (2) a novel approach to assigning time-relevant exposure estimates at the census block level, using all available data on residential mobility throughout the 10-to 20-year period prior to that for which the health events are to be detected.
(iii) We propose an application of this framework using French data to illustrate preliminary results and to describe the feasibility of the different steps of our framework.
(iv) We discuss the need for this conceptual framework, and its subsequent value.

## 2. Retrospective Air Pollution Assessment: An Overview

In this section, we present an overview of assessment approaches of long-term exposure to outdoor pollution that have been developed in epidemiological studies over the past decade. It should be noted that this paper is not intended as a systematic review of modeling approaches and geospatial methods, already available in a previous review [25].

### 2.1. Retrospective Reconstitution of Ambient Air Concentrations and Exposure Assignment

In epidemiological studies, long-term exposure has been characterized by outdoor concentrations at neighborhood or postal addresses whose levels were based on the exposure assignment methods at a central site monitor (or over nearest monitors), or on modeling approaches.

Several studies used only measurements from monitoring stations, some estimating the average of monitors within the study spatial unit [3,12,13,14,23,26], while others used nearest monitor concentrations [2,17,28,29,30], or interpolated values provided by fixed-site monitors [16,24]. Hystad *et al.* combined the spatial patterns detected by original satellite estimates and spatio-temporal patterns from fixed-site monitoring data to estimate ambient air concentrations of NO_2_ for each year from 1975 to 1994 [23]. With a view to assessing exposure to vehicle emissions, Wei *et al.* used State NO_x_ emission data obtained from the air database for 1990 (the earliest year available) to study the incidence of female breast cancer observed during the period 1986–2002 [31], whereas Hystad *et al.* used proximity to the road network to derive the number of years participants had resided within 50, 100 and 300 meters of a highway or major road during the 20-year exposure period [23]. To investigate the long-term effects of urban air pollution in a case-control study of lung cancer in Stockholm, Nyberg *et al.* estimated annual levels of SO_2_ and NO_x_/NO_2_ for each year between 1950 and 1990 using retrospective emission data and linear extrapolation and interpolation [8]. Using a spatial smoothing model and a Geographic Information System, Hart *et al.* estimated annual average levels of PM_10_, SO_2_, and NO_2_ from 1985 through 2000 [2]. Several cohort studies used Land Use Regression (LUR) models to assess small-scale spatial levels of outdoor pollution and assigned exposure for all participants. Using this approach in a Canadian province, Hystad *et al*. calculated an average concentration of NO_2_ for the 1975–1994 exposure period [23]. In the European ESCAPE study, the authors used LUR models to estimate concentrations of NO_2_, PM_10_ and PM_2.5_ in 20 European areas, with 20 sites per area [32,33]. Finally, more sophisticated air dispersion models (such as the CHIMERE chemistry-transport model)—thanks to increasingly available monitoring data and geostatistical modeling—were recently used to retrospectively model outdoor air pollution in France and reconstruct annual average concentrations of NO_2_, PM_10_ and PM_2.5_ from 1989 to 2008 at a fine spatial scale [27].

Hence, most environmental studies characterized exposure levels using outdoor concentration at sites that may be distant to the participants’ residence location. The spatial resolution of the study units varies across these studies: city average level in the Harvard Six Cities Study [3,12,13] as opposed to zip code in the ACS [14,15,16], Medicare national cohort [19] and French Gazel studies [7]. Other spatial scales of exposure assignment were also used, such as district [30,34], enumeration area [35] and census tract [36]. On the other hand, while outcome data and confounder/adjustment variables are available at an individual level, most of these studies, described as being of a semi-ecological design, employ a group-level assessment of exposure.

### 2.2. Retrospective Reconstitution of Cumulative Exposure Levels

When the effects investigated stem from long-term cumulative exposure, one important issue that complicates exposure assessment is accounting for residential changes among the study population [37].

This difficulty of assembling large cohorts and following subjects throughout a long period of time has constrained authors to (i) exclude a large number of participants due to missing residential histories [23] or to select subjects who did not move [38]; or (ii) assign a unique annual exposure to PM_2.5_ and NO_2_ to the cohort members based on the last known home address [2], thus overlooking residential mobility. In some studies, different points in time were used as markers of average air pollution concentrations over the follow-up period, such as the concentration at the address at the study inclusion [39], participant addresses at follow-up [40], or a combination of both [41].

Examples of efforts to reconstitute individual trajectories over decades are the papers by Hystad *et al.*, 2015, who assigned mean exposure levels to traffic-related air pollution from participant residential histories derived for each year over the 20-year period (1975–1994) [23]; and the French Gazel cohort, where the authors assigned annual air pollutant concentrations of PM_10_, PM_2.5_, NO_2_, O_3_, SO_2_, and benzene to participants on the basis of their five-digit zip code [7]. Such efforts are valuable, as recently shown by Andersen *et al.* who evidenced that effect estimates drawn from epidemiological studies were stronger when accounting for residential mobility than when not [42]. Another illustration of the return to taking residential mobility into account is proposed by Gan *et al.* who demonstrated that subjects whose exposure levels had been abated by moving to a less polluted area had a reduced risk of coronary heart disease in comparison to those whose exposure was more constant throughout the entire follow-up period [43].

This residential history issue is more difficult to overcome in ecological studies, where no individual information is available on residential mobility [31]. The following sections propose ways of overcoming this limitation.

## 3. A Conceptual Framework for Retrospective Assessment of Air Pollution

Our framework aims to address the two forms of misclassification described above. To illustrate this framework, we propose ways of retrospectively reconstituting NO_2_ concentrations at the French census block level in the city of Paris (Section 3.1); assigning time-relevant exposure estimates at this spatial scale, using all available data on mobility throughout the 10- to 20-year follow-up period (Section 3.2) and application using data from Paris to illustrate the approach with preliminary results; and to assess the feasibility of the different steps of our framework (Section 4).

### 3.1. Retrospective Modeling of Pollutant Concentrations at a Fine Spatial Scale

To estimate the annual concentrations of NO_2_ since the late 90s at the census block level, a series of three steps is required:

Step 1: Modeling the annual averages of NO_2_ concentrations between 2002 and 2012 at the census block level (the period during which we should have the data for all census blocks);

Step 2: Assessing the spatial area representative of the air quality monitoring stations in the study area;

Step 3: Modeling the temporal trend of: (i) annual averages between 2002 and 2012 at the census block level; (ii) and of daily NO_2_ concentrations obtained from the monitoring stations, and subsequently reconstructing the annual averages before 2002 at the census block level with this data.

#### 3.1.1. Step1: Modeling the Annual Averages of NO_2_ Concentrations between 2002 and 2012 at the Census Block Level

Annual mean concentrations of NO_2_ were estimated at a fine spatial scale (IRIS) throughout the 2002−2012 period by Airparif (the Paris metropolitan area air quality monitoring network: http://www.airparif.fr) [44].

Firstly, NO_2_ background concentrations were determined by combining monitored NO_2_ concentrations from monitoring stations and those modeled at a regional scale from the ESMERALDA inter-regional platform for air mapping and forecasting (www.esmeralda-web.fr). The ISATIS software was used to conduct geostatistical analysis for data assimilation. Secondly, NO_2_ road traffic concentrations estimated from the STREET software model [45] were added to NO_2_ background concentrations. The software evaluates the annual levels from roads according to traffic characteristics and the close environment, as well as weather conditions. Several types of input data were used, including point sources and road transport emissions and meteorological data (temperature, wind speed and direction, relative humidity, barometric pressure). More than 200 point sources were selected from the regional emission inventory. Emissions for road traffic were estimated using the regional traffic network and the COPERT III European database for the 2002–2006 period, and COPERT IV for the 2007–2012 period. Concerning meteorological data, the Mesoscale Meteorological model (MM5: www.mmm.ucar.edu/mm5) developed by the Division of the NCAR Earth System Laboratory (NESL) was used. The areas of direct influence of the axes and the decreasing concentrations away from the latter are taken into account. This decrease is estimated through measurements on sites influenced by road traffic.

#### 3.1.2. Step 2: Assessing the Spatial Area Representative of the Air Quality Monitoring Stations

Using daily NO_2_ concentrations measured by fixed monitoring stations (including background stations and traffic stations) located within the city of Paris and available over the 2002–2012 period, we identified the area for which the air quality monitoring stations provided good estimates of daily concentration variability.

The hierarchical agglomerative clustering (HAC) is the most commonly used approach as it provides intuitive similarity relationships between any one sample and the entire dataset. The HAC was chosen and applied for Paris to associate each census block with a background permanent monitoring station. This cluster analysis allowed grouping census blocks and stations into clusters (groups) on the basis of similarities within a class and dissimilarities between different clusters.

Each census block was then assigned by Airparif to the monitoring station (named the “index” monitor) best representing overall NO_2_ air quality within the census block, using clustering methods [46].

#### 3.1.3. Step 3: Reconstitution of the Retrospective Annual Averages Prior to 2002 at the Census Block Level

In this final step, we combine the concentrations measured by monitoring stations with those estimated at the census block level for the associated “index” monitor. First, we assess temporal trends of both the daily concentrations measured at the Paris monitoring stations and the annual concentrations estimated at census blocks during the study period (2002–2012). Thus, the NO_2_ variability (daily at each “index monitoring” level, and annually at each census block level) will be modeled using time-series analysis.

Second, we suggest weighting the annual average for the census block using the coefficient resulting from the time-series analysis of its “index” monitor. In other words, the calculations of retrospective NO_2_ concentrations have two components: a census block component corresponding to the NO_2_ annual trend, and a local component “index” monitor. Therefore, we use the daily trend coefficient to weight the annual observations.

### 3.2. Assessment of Cumulative Exposure Accounting for Residential Mobility

**Figure 1:**
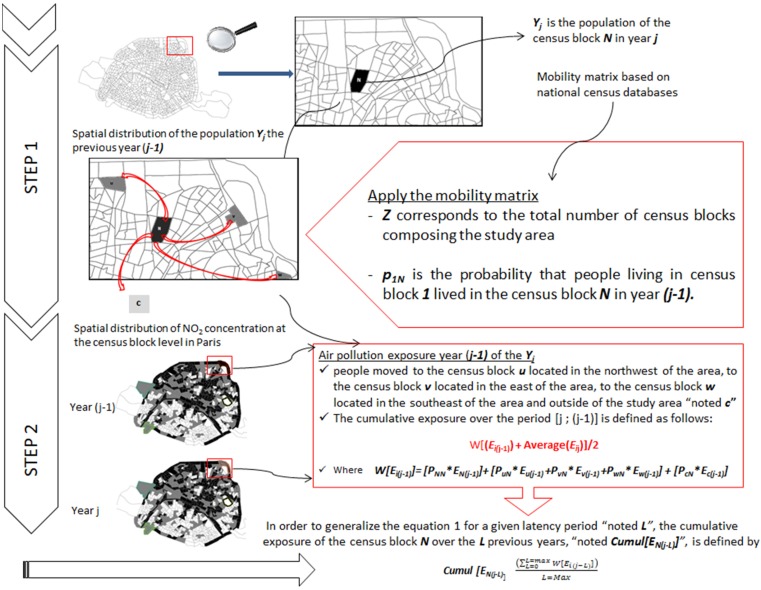
Assessment of cumulative exposure accounting for residential mobility.

Figure 1 describes the two main steps required to fully assess cumulative exposure over the disease’s long latency period. This framework explains how residential mobility can be integrated as a correction factor to retrospectively estimate cumulative exposure at the census block level.

- First, we retrieve the information from the national census that describes the proportion of the residents of each census block that lived in another census block in the past.
- Second, we derive a population cumulative exposure estimate by weighting the average ambient air levels of the various census blocks according to the population that resided in other census blocks (or cities) in the past.

#### Theoretical Model

— Let N denote the census block with an average exposure level ENj in the year j.
— Let us define U, V and W census blocks comprising the study area with the corresponding average exposure level Euj, Evj and Ewj, estimated in the year j.
— Let C denote the area outside the study area with Ecj, the corresponding average exposure level estimated in the year j.

Under the hypothesis that people living in census block ***N*** in year ***j*** could have lived in another census block one year previously, one can combine exposure levels for the different places of residence (namely ***U***, ***V***, ***W*** and ***C***, in our present example) into a single estimate.

Using a mobility matrix, constructed from the national census database, describing inhabitant movements intra-area and outside the study area (*i.e.*, another municipality, department or region) from one year to a previous year, the model estimate’s cumulative population exposure is an average measure between ***E_N_***_j_ and the weighted [***E_i(_*_j-1)_**] where ***i*** represents the census blocks of residence in the ***(j-1)*** year. In our example, ***i*** can be the census block ***U***, ***V***, ***W*** and ***C***.

The weighted [***E_i(_*_j-1_**_)_] (noted *W*[***E_i(_*_j-1_**_)_] in following text) is defined according to the following equation:

W[E_i(__j-1)_] = [P_NN_*E_N(__j-1)_] + [P_uN_*E_u(__j-1)_ + P_vN_*E_v(__j-1)_ + P_wN_*E_w(__j-1)_] + [P_cN_*E_c(__j-1)_] (1)

where:

- [P_NN_* E_N(__j-1__)_] characterizes the sedentary population; ***p_NN_*** is the probability that the population residing in census block ***N*** already lived in the same census block ***N*** in year ***(j-1)***;
- [P_uN_*E_u(__j-1)_ + P_vN_*E_v(__j-1)_ + P_wN_*E_w(__j-1)_] characterizes the intra-area movement of the population;
  ○ ***p_uN_ p_vN_*** and ***p_wN_*** are probabilities that the population residing in census block ***N*** lived, respectively, in the census block ***U, V or W*** in year ***(j-1)*** and
  ○ ***E_u(__j-1)_, E_v(__j-1)_ and E_w(__j-1)_*** are, respectively, the average exposure level of census block ***U, V or W*** in year ***(j-1)***
- [P_cN_*E_c(__j-1)_] characterizes the population movement outside the study area;
  ***p_CN_*** is the probability that the population residing in census block *N* lived in location ***C*** (municipality or department or region) in year ***(j-1)***
  ○ ***E_c(__j-1)_*** is the average exposure level of the location ***C*** in year ***(j-1)*** extracted from the meta-analysis of French studies conducted at the municipal and regional scales (e.g., Bentayeb *et al.,* 2014).

From Equation (1), we deduct that a low level of mobility for census block ***N*** is quantified by a probability ***P_NN_***, close to 1 (approximately *100% of inhabitants live in census block **N** in year **j** and in the year **(j-1)***) and as a consequence, others ***P_uN_***, ***P_vN_***, ***P_wN_*** and ***P_cN_*** will be near to 0 (and *vice versa).* Between years ***(j-1)*** and ***(j-2)***, Equation 1 is again applied for each new location, ***N***, ***U***, ***V***, ***W*** and ***C*** to assess exposure by taking into account population mobility *etc.* across the latency duration of the disease of interest.

In order to generalize Equation (1) for a given latency period designated as ***L***, the cumulative exposure of census block ***N*** over the ***L*** previous years, designated as ***Cumul [E_N(j-L)_]***, is defined according to Equation (2):

(2) $$Cumul[E_{N\left(j-L\right)}]=\frac{\sum_{L=0}^{L=max}W\left[E_{i\left(j-L\right)}\right]}{L=max}$$

where the numerator is calculated from Equation (1).

## 4. An Application Using Data from Paris

### 4.1. Study Setting

The city of Paris (Ile-de-France region, capital France) is subdivided into 20 “arrondissements” and 992 census blocks for a total population of approximately 2,250,000 inhabitants. Designed by the French National Census Bureau (INSEE), the census block named “IRIS” constitutes the smallest census unit area whose aggregated data can be routinely used.

However, some data were available only at the arrondissement level when this analysis was undertaken (e.g., residential mobility data). Therefore, in this first application, some steps of our framework were carried out only at the arrondissement level, to illustrate the approach.

### 4.2. Step1: Annual NO_2_ Concentrations between 2002 and 2012 at the Census Block Level

**Figure 2:**
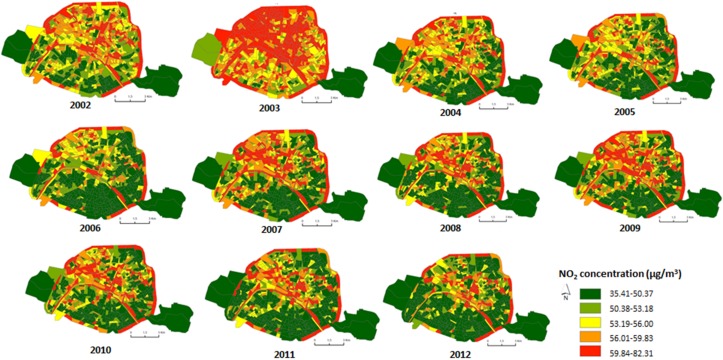
Spatiotemporal distribution of the modeled annual averages of NO_2_ concentrations at the census block level over the 2002−2012 period in Paris.

**Figure 3:**
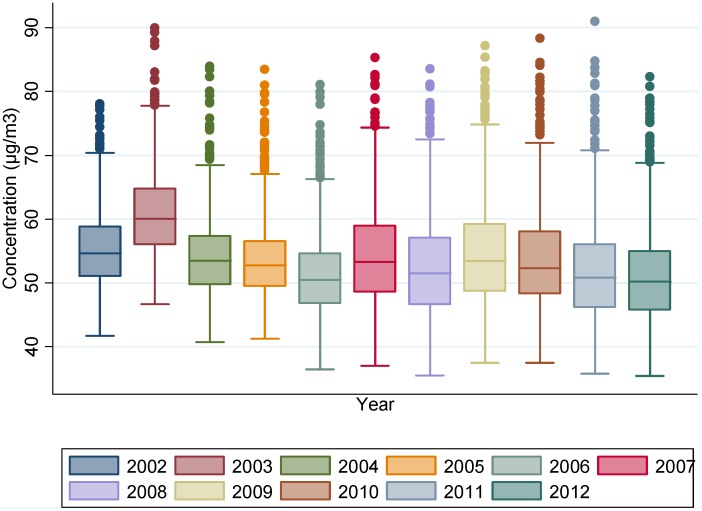
Distribution of NO_2_ concentrations at the census block level (in µg/m^3^) from 2002 to 2012. Box plots (fifth percentile, first quartile, median, third quartile, ninety-fifth percentile).

Results obtained from this first step showed a regular decrease trend in NO_2_ concentrations at the census block level between 2002 and 2012, with 61.0 µg/m^3^ in 2003 and 51.2 µg/m^3^ in 2012 (Table 1). Higher NO_2_ annual means were localized in the northern part of Paris. The spatio-temporal trend is given in Figure 2 that exhibits the annual concentrations of NO_2_ between 2002 and 2012, using the dispersion model at a 2.5 km resolution. Figure 3 summarizes the NO_2_ concentrations for each year at the Paris city scale, showing a high between-census block variability regardless of the year. For instance, in 2002, the minimum NO_2_ concentration at the census block level was 41.8 µg/m^3^ while the maximum was 78.0. The same pattern was observed in 2012 with the lowest NO_2_ concentration at 35.4 µg/m^3^ and the maximum at 82.3.

**Figure 4:**
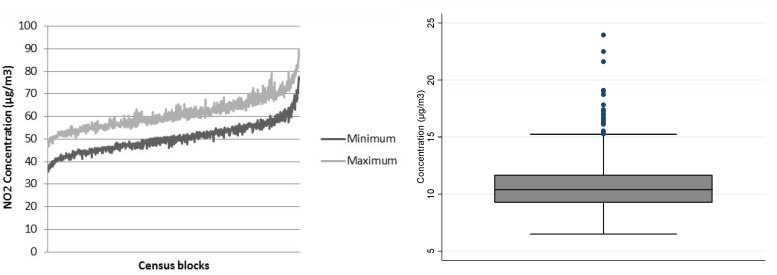
Distribution of intra-census block variability (difference between maximum and minimum of NO_2_ concentrations at the census block level (in µg/m^3^)) for the entire study period from 2002 to 2012. Box plots of the difference between the maximum and the minimum (fifth percentile, first quartile, median, third quartile, ninety-fifth percentile).

The distribution of NO_2_ annual concentrations across all census blocks expressed as (min; max) values are shown in Figure 4 where the X axis exposes the census blocks ordered by ascending value of the NO_2_ census block mean between 2002–2012.

While the between-census block variability of annual average NO_2_ concentrations was high (Table 1 and Figure 3), Figure 4 shows that the intra-census block variability was low. It also reveals that the difference between the maximum and the minimum of the annual average of NO_2_ concentrations is stable over all census blocks, from those with low NO_2_ concentrations (40.0 µg/m^3^) to those with high NO_2_ concentrations (83.3 µg/m^3^) with a common value about 10 µg/m^3^ ((35.5–46.7) and (78.7–89.9), respectively).

### 4.3. Step 2: Assessment of the Spatial Representativity of the Air Quality Monitoring Stations

**Figure 5:**
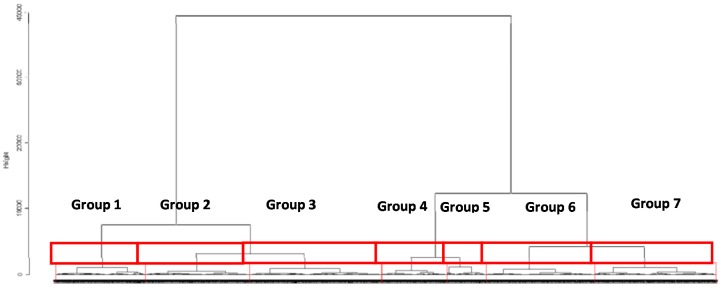
Dendogram of distances between all census blocks and monitoring stations.

This second step reveals seven groups of census blocks and their associated monitoring stations according to their typology (urban, peri-urban and traffic monitoring stations). The dendrogram (Figure 5) provides a visual summary of the clustering process, presenting a picture of the groups and their proximity.

Using the groups defined by the AHC, we chose the best representative air quality monitoring station for each census block on the basis of their spatial proximity (if we had two stations in the same cluster). Descriptive statistics of air quality monitoring stations are presented in Table 2.

### 4.4. Step 3. Illustration of Time Trends during the Study Period (2002–2012)

To give an illustration of the process followed in step 3, we chose the three representative stations of census block groups 4, 5 and 7 in order to describe the variability of daily concentrations measured by traffic and urban monitoring stations (named N2BONA, N2PA07, N2AUT). For each station, we selected three census blocks among all in the corresponding groups for which the station was representative of their NO_2_ daily variations (N2BONA: census block A, B, C; N2PA07: census block D, E, F and N2AUT: census block G, H, I).

**Figure 6:**
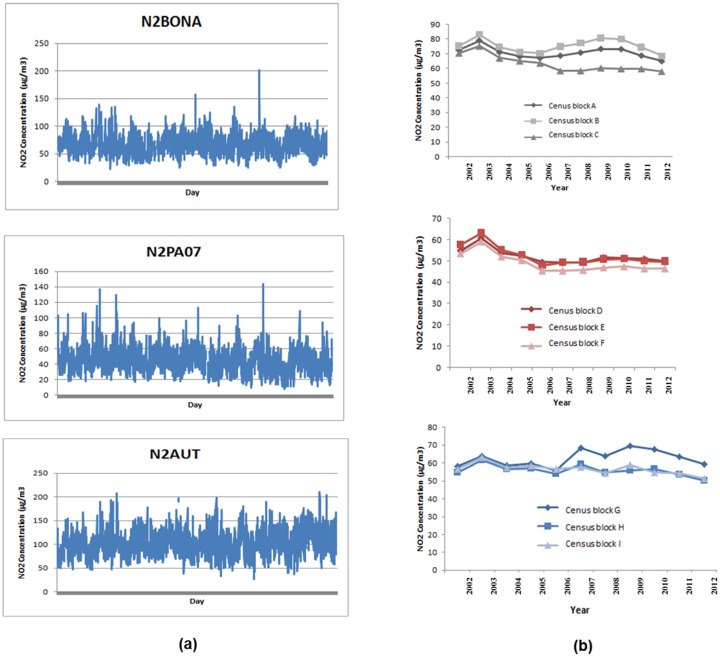
Illustration of time trends during the study period (2002–2012): (**a**) the variability of daily concentrations measured at three monitoring stations named the “index” monitors; (**b**) the variability of annual concentrations estimated for three census blocks among all the census blocks represented by each monitoring station.

Figure 6 reveals that even if census blocks were classified in the same group (defined by step 2), the trends of NO_2_ annual concentrations differ between census blocks during the study period (2002–2012); one could have a linear trend whereas others could not be described by linear trends. For instance, the three census blocks of group 5 (A, B and C) have the same trend until 2005, whereas beyond this group each census block has its own trend. Inversely, in group 7, the three census blocks (D, E and F) exhibit similar trends over the study period. In group 4, the census blocks follow the trend during the beginning of the study period. However, after 2006, one census block tends to differ from the two others.

These observations confirm that within the same group of census blocks for which one monitoring station has been identified to best represent the NO_2_ daily variability, considering only the daily NO_2_ concentrations of the monitoring station (named “index monitoring”) will provide retrospective good annual average estimates of NO_2_ concentrations at the census block level. A specific time-series analysis is necessary to assess the temporal trends and estimate the specific coefficient relating each “index monitoring” and each census block.

### 4.5. Estimation of the Cumulative Exposure Accounting for Residential Mobility

**Figure 7:**
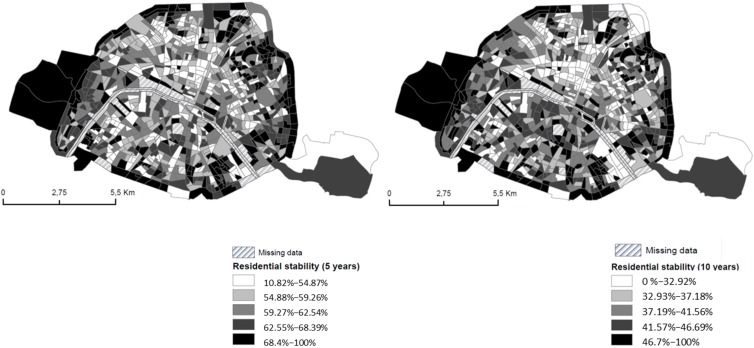
Spatial distribution of the proportion of people residing in 2006 in census block and living in the same census block 5 and 10 year before.

To take into account residential mobility (the P_NN_, P_uN_, P_vN_ and P_wN_, notations in Section 3.2.1.); we used a database extracted from the 2006 National Census by INSEE (Institut National de la Statistique et des Etudes Economiques). The spatial distribution of the proportion of people living in 2006 since for five or ten years in the same census block in Paris is presented in Figure 7: in half of the census blocks, 60% and 40% of people living in 2006 in a given census block resided in the same place five and ten years before, respectively. Unfortunately, because INSEE currently provides data of residential moves within Paris at the arrondissement and not census block level, we applied our theoretical approach (described in Section 3.2.1) at this larger spatial scale for a five-year latency period (the L in our notations, Section 3.2.1.).

Using a mobility matrix (see Supplementary Materials Figure S1), constructed from the national census database, we determined for each arrondissement the number of inhabitants changing their arrondissement of residence over the last five years (the arrondissement where people lived five years earlier) and the origins of the inhabitants from another arrondissement. In our example, we only consider population movements inside the study area, ignoring at this stage movements “outside” (movements to or from other cities) (see the Matrix in Supplementary Materials Figure S1).

Table 3 gives the cumulative NO_2_ exposures over five years by arrondissement with and without taking into account residential movements inside the study area. The arrondissements are classified in descending order of the degree of residential mobility. The results reveal that when including the mobility matrix in the estimation of cumulative NO_2_ exposures, population exposure levels were always lower than when not taking into account the residential mobility, the difference increasing with the mobility degree. For example, comparing arrondissement number 1 (with the lowest mobility rate, equal to 8.8%) with arrondissement number 15 (the highest mobility rate, equal to 19%), the differences of NO_2_ cumulative exposure estimates with and without taking into account residential mobility are equal to 3 and 6 µg/m^3^ respectively.

Table 3 shows the relative difference between these two exposure levels (with and without residential mobility). The relative difference across the 20 arrondissements varies between 2.7 to 5.6 µg/m^3^. It is this difference, expressed in µg/m^3^, which is attributed to residential mobility and may change the result of the epidemiological studies; it may amount to up to 13% of average exposures and is likely to be greater when we are able to proceed to this analysis at the census block level.

## 5. Discussion and Perspective

**Figure 8:**
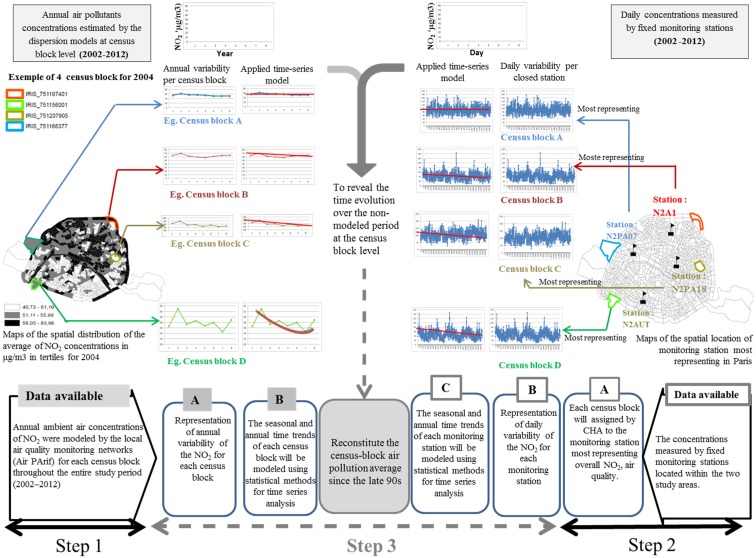
Retrospective modelling of pollutants’ concentrations at a fine spatial scale.

This framework is designed to overcome limitations regarding exposure assessment to air pollution at a fine spatial scale in epidemiologic studies investigating long-term health effects. The conceptual model summarized in Figure 8 comprises a set of three steps. This approach comes into its own when pollutant concentration variations between census blocks are greater than those within. Using French data, the application of steps 1 and 2 of this conceptual framework reveal that specific and complex time-series analysis is necessary to the assessment of temporal trends and estimation of the specific coefficient relating to each “index monitoring” and each census block. The third step (which is sophisticated and elaborate, and not within the scope of this paper) must be specific to each study, its context and its design.

Moreover, the illustration of the estimation of cumulative exposure accounting for residential mobility at arrondissement level has revealed significant differences between estimations with and without residential mobility over a five-year period. However, the mobility matrix must be completed at the census block level in order to combine this approach with the retrospective constitution of NO_2_ concentrations at the census block level. To overcome the fact that residential mobility data is not currently provided at the census block level, different apportioning solutions are available for the disaggregation of population data from arrondissement to census block level via the combination of several data sources, including topographic and land use databases.

A major limitation of our method is that it remains relatively cumbersome, labor-intensive, and computer-intensive, requiring extensive data inputs that are generally difficult to obtain. Nevertheless, this conceptual model strives to address the following two aspects.

Firstly, the retrospective construction integrates models assessing pollutant concentrations at 25 m^2^ resolution scales, whereas up until now, the spatial resolution of previous approaches was limited to coarser spatial resolution levels: 10 km^2^ [23], 2 km^2^ [7,27], 1 km^2^ [47], 200 m^2^ [48] and 100 m^2^ [8]. Other models with similar spatial performance can be used along the same lines. This conceptual framework was designed for ecological approaches, as in the study we are undertaking on breast cancer, but we are confident that the concept is adaptable to other study designs; for instance, examining annual air pollution concentrations of cohort participants’ census blocks rather than zip codes [7]. We illustrated the conceptual model on NO_2_ but the same rationale can be applied to other pollutants such as PM_10_ or PM_2.5_ after adapting input data (including background pollution measurements and monitoring station measurements) accordingly.

Secondly, we propose taking residential mobility into account in the cumulative exposure assessment (using the mobility coefficient described in our framework). This is a crucial point in considering a disease with a long latency period, such as cancer. Therefore, the value of this approach lies beyond ecological, cohort and case-control studies with information on residential history, and may also be useful for enabling more accurate reconstruction of cumulative exposure.

We have also identified further limitations to this framework, mainly relating to uncertainties due to (a) exposure assignment method and (b) cumulative exposure assessment.

First, uncertainty around retrospective modelled concentration stems from the spatio-temporal variability of the model input parameters which depend on dispersion factors (both meteorological and topographical characteristics) and emission inventories, as well as on the model formulation itself. The robustness of the dispersion model integrated in our framework has, however, been thoroughly evaluated and validated in previous studies [46,49,50]. Yet this strength also has disadvantages, since the atmospheric dispersion models are complex and require data-demanding software that may be difficult to implement—even impossible in some countries, due to lack of input data.

In addition, in areas with a small number of monitoring stations during the first years of the study period (such as in our case during the 1990s), the possibility of retrospective reconstitution of air pollution concentrations may be limited. Assessment of the extent of errors made for retrospective estimates of ambient air concentrations may be carried out for a limited number of pollutants using information from monitoring sites that existed for the same pollutants in the past.

A second limitation relates to the intensity of residential mobility. In areas where population size and/or socio-demographic make-up has changed markedly during the time span of the epidemiological study, the method we propose will carry substantial uncertainty that will be greater in census blocks with a high degree of mobility, in comparison to sedentary census blocks.

In the next stage, we envisage an extension of our framework by also considering the daily mobility associated with occupational mobility. An alternative perspective is to evaluate the impact of the error introduced by the multiple steps of the estimation process on retrospective reconstitution of air pollution concentrations estimated at the census block level.

To achieve this, we will apply the retrospective method to other years—those for which the dispersion model has already provided validated estimates at the census block level. For instance, using data from 2005 to 2012, we will estimate retrospective concentrations for the period 2002 to 2004. Then, we will quantify the margins of error inherent to our approach, by estimating the error between the concentrations retrospectively obtained, and the modelled NO_2_ concentrations.

## 6. Conclusions

In order to explore the health effects of chronic exposures, we seek to combine several sources of data routinely collected and modeled so as to address the accuracy of cumulative estimates of NO_2_ concentrations over periods of years and, based on these, on population exposure, taking into account residential mobility over time, its duration being characterized according to the latency delay for development of the disease. Our framework explains how to surmount two principal difficulties in cumulative long-term exposure assessment: retrospective reconstitution of past ambient air concentrations and consideration of residential mobility in assigning exposure levels to neighborhoods; and, as appropriate according to study design, to individuals. We have provided guidelines on how census database and geostatistical methods may inform an approach for correcting the above-described sources of cumulative exposure misclassification. The conceptual framework is flexible and convenient for the needs of different epidemiological study designs. As a domain of application for future work, it would be interesting to explore the impact of the above-described misclassification on assessing the relationship between long-term exposure and cancer risk.

## Figures and Tables

**Table 1:** Descriptive statistics of NO_2_ concentrations across the study period (2002–2012) for Paris.

Statistical Indicators	Year										
	2002	2003	2004	2005	2006	2007	2008	2009	2010	2011	2012
Mean *	55.34	60.96	54.22	53.52	51.14	54.25	52.59	54.60	53.70	52.01	51.17
Standard Deviation	5.96	6.63	6.20	5.82	6.23	7.72	7.86	8.07	7.79	7.93	7.48
Median *	54.63	60.02	53.52	52.74	50.45	53.27	51.54	53.43	52.31	50.82	50.19
Minimum *	41.75	46.68	40.73	41.23	36.43	37.01	35.50	37.50	37.50	35.75	35.41
Maximum *	78.03	89.93	83.96	83.43	81.05	85.31	83.53	87.14	88.33	90.98	82.31

* expressed in µg/m^3^.

**Table 2:** Descriptive statistics of air quality monitoring stations in Paris.

Type of Monitoring Station	Name of Station	Mean *	EC	Total of Census Block
Urban	N2PA06	38.51	13.81	1
Urban	N2PA07	41.35	14.57	316
Urban	N2PA12	46.83	12.26	33
Urban	N2PA13	40.75	12.05	1
Urban	N2PA18	45.64	17.49	283
Traffic	N2A1(AutA1)	90.22	18.07	21
Traffic	N2BONA	66.38	11.476	69
Traffic	N2AUT (BPAUT)	100.39	24.70	29
Traffic	N2BASC	91.63	19.09	45

* expressed in µg/m^3^.

**Table 3:** Population average exposure levels to NO_2_ over 5 years at the arrondissement level with and without considering residential mobility (arrondissements are ranked according to the intensity of the between-census mobility)

Arrondissement	Degree of Mobility (%)	Cumulative Exposure without Residential Mobility *	Cumulative Exposure with Residential Mobility *	Relative Difference *
Arrondissement 15	19.0	44.23	38.64	5.59
Arrondissement 17	18.5	49.91	44.00	5.91
Arrondissement 14	18.4	42.17	37.75	4.42
Arrondissement 11	18.4	45.74	41.14	4.60
Arrondissement 10	18.3	48.15	43.76	4.39
Arrondissement 9	17.1	46.99	43.32	3.66
Arrondissement 16	17.0	46.10	40.81	5.29
Arrondissement 18	16.5	47.56	42.89	4.67
Arrondissement 5	16.2	44.60	40.66	3.94
Arrondissement 2	15.8	46.64	43.51	3.13
Arrondissement 3	15.6	49.10	45.55	3.55
Arrondissement 8	15.4	48.00	44.46	3.54
Arrondissement 7	15.4	44.60	40.42	4.18
Arrondissement 12	15.3	46.09	42.30	3.78
Arrondissement 13	14.6	41.07	37.94	3.13
Arrondissement 20	14.1	45.51	42.46	3.05
Arrondissement 6	13.1	45.84	42.07	3.77
Arrondissement 4	12.7	49.17	45.67	3.50
Arrondissement 19	12.6	45.91	43.20	2.71
Arrondissement 1	8.8	50.46	47.34	3.11

* expressed in µg/m^3^.

## Supplementary Materials

The following are available online at www.mdpi.com/1660-4601/13/3/319/s1, Figure S1: Description of the population movement inside the study area.
