# Supplementary material for: A Conceptual Framework for the Assessment of Cumulative Exposure to Air Pollution at a Fine Spatial Scale
Source: Int J Environ Res Public Health. 2016 Mar 15;13(3):319. doi: 10.3390/ijerph13030319 (PMC4808982; doi:10.3390/ijerph13030319)
Supplement: Supplementary file 1 [file ijerph-13-00319-s001.pdf]

Supplementary Materials: A Conceptual Framework for the Assessment of Cumulative Exposure to Air Pollution at a Fine Spatial Scale

Wahida Kihal-Talantikite, Cindy M. Padilla, Denis Zmirou-Navier, Olivier Blanchard, Geraldine Le Nir, Philippe Quenel and Séverine Deguen

Mobility matrix	arrondissement10	arrondissement11	arrondissement12	arrondissement13	arrondissement14	arrondissement15	arrondissement16	arrondissement17	arrondissement18	arrondissement19	arrondissement1	arrondissement20	arrondissement2	arrondissement3	arrondissement4	arrondissement5	arrondissement6	arrondissement7	arrondissement8	arrondissement9	Total
**arrondissement10**		1175	393	365	355	464	281	483	1203	712	126	719	382	552	220	229	180	117	112	667	8735
**arrondissement11**	1043		1355	888	635	984	422	507	875	866	197	1433	513	613	635	586	336	204	123	438	12653
**arrondissement12**	447	1822		1122	499	807	317	452	619	770	121	1412	120	206	279	584	165	189		170	10101
**arrondissement13**	503	1086	885		1405	1267	470	492	768	822	118	930		184	236	1226	244	313	158	151	11258
**arrondissement14**	355	511	490	1195		1850	596	516	619	485	109	631		153	121	722	743	445	156	220	9917
**arrondissement15**	492	743	795	1182	2091		1853	1133	913	660	144	622		254	191	654	832	1039	379	467	14444
**arrondissement16**	250	288	328	427	545	1937		1122	402	353	115	273		142	132	299	335	787	628	251	8614
**arrondissement17**	378	727	526	549	608	1126	1400		1948	750	101	574	187	304	126	264	163	371	866	666	11634
**arrondissement18**	953	1304	641	765	584	1002	522	1983		1272		1283	236	299	174	407	167	222	260	1121	13195
**arrondissement19**	1547	1610	456	687	422	715	419	763	1996			2189	185	324	205	276	113	123		409	12439
**arrondissement1**		171						108					119			106					504
**arrondissement20**	1028	2844	1360	828	468	692	310	768	1960	2142			187	384	142	334	136	155		376	14114
**arrondissement2**	135	209	110	123		140	112	175	204		185	145		144	121		103				1906
**arrondissement3**	256	310	212	122	124	186	162	197	149	172	118	187	175		309	133	135			127	3074
**arrondissement4**		206	138	135	106	180	137	172	121	155				155		106	130				1741
**arrondissement5**	187	259	204	707	448	331	263	209	217	224		145		205	214		483	261		134	4491
**arrondissement6**		111		205	331	432	341	184								317		360			2281
**arrondissement7**		131		165	189	866	521	284	125			135				195	508		228		3347
**arrondissement8**	163	100				286	564	848	192	106		131				103	157	256		309	3215
**arrondissement9**	453	358	173	203	226	418	376	630	682	241	127	240	165	196	104	160	232	153	316		5453
**Total**	8190	13965	8066	9668	9036	13683	9066	11026	12993	9730	1461	11049	2269	4115	3209	6701	5162	4995	3226	5506	153116

Figure S1. Description of the population movement inside the study area*. Note:* The mobility matrix describes the number of people residing in a given arrondissement in 2006 (in column) who resided in other arrondissements 5 years before (in line).

© 2016 by the authors; licensee MDPI, Basel, Switzerland. This article is an open access article distributed under the terms and conditions of the Creative Commons by Attribution (CC-BY) license (http://creativecommons.org/licenses/by/4.0/).
